# Induction of labour versus expectant management after one previous caesarean birth maternal and neonatal outcomes in a population-based cohort study

**DOI:** 10.1038/s41598-026-70966-9

**Published:** 2026-09-09

**Authors:** Susanne Hesselman, Johanna Gunnarsdóttir, Erik Lampa, Anna-Karin Wikström, Sophia Brismar Wendel, Maria Jonsson

**Affiliations:** 1 Department of Women’s and Children’s Health, Uppsala University, Uppsala, Sweden; 2 Faculty of Medicine, University of Iceland, Reykjavik, Iceland; 3 Epistat AB, Uppsala, Sweden; 4 Department of Women’s and Children’s Health, Karolinska Institutet, Stockholm, Sweden; 5 Medical Unit Pregnancy and Childbirth, Women’s Health and Allied Health Professionals Theme, Karolinska University Hospital, Stockholm, Sweden; 6 Centrum för Klinisk Forskning Dalarna, Region Dalarna, Falun, 791 29 Sweden

**Keywords:** Caesarean section, Vaginal birth after caesarean, Obstetric labour complications, Uterine rupture, Neonatal diseases, Diseases, Health care, Medical research, Risk factors

## Abstract

To investigate maternal and neonatal outcomes after induction of labour (IOL), across all indications, compared with expectant management at term among women with prior caesarean section (CS). Women with one prior CS planned for vaginal birth 2007–2020 (N = 38 689). IOL was investigated as a continuum in days and compared with expectant management (EM; onset of birth one day or more later). The risk of severe and moderate maternal and neonatal outcomes at gestational week 37–42 was compared between IOL and EM using a multivariable logistic regression model. Results are presented as adjusted absolute risks, relative risks, and adjusted risk differences (aRD), with 95% confidence intervals (CIs). IOL compared to EM increased the risk of CS with the maximum at 40 weeks (aRD 5.5%, 95% CI 3.0;6.6). The risk of severe maternal and neonatal outcomes increased with gestational age (p < 0.001), without difference between IOL and EM. Moderate severe neonatal outcomes were increased at week 37 (aRD 3.8, 95% CI 1.4;7.4) but reduced with IOL at week 39 (aRD − 1.0, 95% CI -1.6;-0.3). For women with prior CS, IOL at weeks 39–41 was associated with unplanned CS. Timing of birth influence neonatal outcomes.

**Supplementary Information:**

The online version contains supplementary material available at https://doi.org/10.1038/s41598-026-70966-9.

## Introduction

It is well established, from observational studies and clinical evidence, that women attempting a vaginal birth after caesarean section (CS) are at risk of maternal and neonatal birth complications^1–5^. Women with a history of CS are frequently excluded from randomized controlled trials (RCTs) investigating intrapartum interventions. Nevertheless, evidence from these trials is often extrapolated to guide obstetric practice for all births irrespective of prior mode of birth.

Induction of labour (IOL) is a common medical intervention in modern obstetrics, intending to improve neonatal or maternal outcomes^6^. For example, compared with expectant management, IOL late preterm to early term improves maternal health among women with hypertensive disorders of pregnancy^7,8^ and IOL at 39 weeks in low-risk pregnancies reduces the risk of CS but neither improve nor deteriorate neonatal health^9^, while IOL at 41 weeks compared with expectant management until week 42 reduces perinatal mortality and morbidity^10,11^. In only one of these clinical trials, women with prior CS were invited to participate^8^, indicating the lack of evidence for these individuals, regardless of IOL for medical and non-medical conditions.

Few RCT: s has investigated IOL compared with expectant management in women with prior CS. An RCT from 1999 (*n* = 294) comparing induction with prostaglandins with expectant management at term in women with prior CS found no increased risk of adverse maternal outcomes or repeat CS with IOL^12^. In observational studies, IOL among women with prior CS has been associated with maternal and perinatal morbidity^4,5,13–15^. Apart from confounding by indication, the comparison with spontaneous onset at a particular time point introduces bias, since the assignment to onset of labour can only be IOL or expectant management. Moreover, maternal and neonatal outcomes are often reported by actual mode of birth and not intended mode of birth, limiting the clinical use in consultation and decision of timing of labour^1,4^.

With this study, we aimed to mimic a clinical situation and investigate IOL at different gestational weeks on maternal and neonatal outcomes compared with expectant management, by using prospectively collected data from a nationwide birth register. Women were considered assigned to IOL, irrespective of the indication, on the gestational day they gave birth following IOL, or to expectant management if the date of birth occurred one or more days later, regardless of whether onset of labour was spontaneous, induced, or by unplanned CS.

## Methods

One randomly selected singleton birth per woman, 2007–2020, was identified in the Swedish Medical Birth Register (MBR), and from that cohort women with prior CS was identified.

Women with births registered as elective CS, more than one prior CS or unknown number of prior CS (i.e., numbers not recorded in MBR), placenta previa, placenta accreta, preterm pre-labour rupture of membranes, or gestational length < 37 weeks or congenital anomaly were excluded. Medical information was retrieved from electronic birth records, including information from checkboxes and diagnostic codes according to the Swedish version of the 10th revision of the International Classification of Diseases (ICD-10) at discharge from the hospital. Diagnostic codes within 42 days from birth were also retrieved from the Cause of Death Register, as well as the national Patient Register, including diagnoses from all inpatient and outpatient specialized care.

Onset of birth (spontaneous, induction, or pre-labour CS) was based on manually entered midwife-reported checkboxes in the electronic birth records forwarded to the MBR and in addition the diagnostic code O61^16^. The intervention studied was birth after IOL at a specific date versus birth later, i.e., expectant management. Births with spontaneous onset or by CS that occurred the same day as induced births were censored that specific day. Expectant management was thus defined as birth one day or more after the date, regardless of onset (spontaneous, induction, or prelabour CS). Gestational age was determined in 92.4% of pregnancies by first- or second-trimester ultrasound, in 3.6% on the date of the last menstrual period, and in 3.9% through postnatal assessment.

Maternal demographics and characteristics included age at birth (years), manually entered information at first antenatal visit of height (cm), weight (kg), country of birth classified as Nordic country or non-Nordic (other European country/North America, Asia, Africa or other), civil status (cohabitant yes/no), and educational attainment (≤ 12 and > 12 years). Pre-gestational disorders retrieved from checkboxes (yes/no) included any of the following self-reported conditions: hypertension, diabetes mellitus, epilepsy, inflammatory bowel disease, systemic lupus erythematosus, kidney disorder, and prior vaginal birth, irrespective of numbers.

Interpregnancy interval was months from the preceding birth (year-month) to the estimated timepoint of conception (calculated by subtracting gestational age at birth in weeks and days from year-month of birth and then adding two weeks). Gestational disorders were defined by diagnostic codes (ICD-10) during pregnancy or at discharge including gestational hypertension (O13), preeclampsia (O11, O14-16), and gestational diabetes (O244, O249). Neonates small (SGA) or large (LGA) for gestational age according to estimated neonatal weight curves were used as a proxy for intrauterine growth restriction (−2SD SGA) or macrosomia (+ 2 SD LGA)^17^.

Investigated outcomes were based on core outcomes for IOL proposed by Dos Santos et al^18^, and decided a priori. The primary outcome was mode of birth with unplanned pre-labour or intrapartum CS as opposed to vaginal birth, including spontaneous, vacuum extraction, or forceps. This outcome was stratified for women with prior vaginal birth and no prior vaginal birth, and for women with and without pre-gestational and gestational disorders. Secondary outcomes were four composite outcomesSevere maternal outcome including maternal death within 42 days from birth, re-operation within 42 days from birth, or uterine rupture (O710, O711) Moderate maternal outcome including blood loss > 1000 ml at birth or a diagnosis of anaemia at discharge, obstetric anal sphincter injury (OASI) recorded by check-boxes, or postpartum wound infection, hematoma, or dehiscence diagnosed within 42 days from birth Severe neonatal outcome including stillbirth or neonatal death within 28 days from birth, neonatal birth trauma (P10-14), cerebral haemorrhage (P52), seizures (P90), hypoxic ischemic encephalopathy and other cerebral disturbances (P91), necrotizing enterocolitis (P77), or an Apgar score < 4 at five minutes Moderate neonatal outcome including neonatal infection (P23), respiratory distress syndrome or neonatal aspiration (P22, P24), or Apgar score < 7 at five minutes.

Additional secondary outcomes were two individual maternal outcomes: uterine rupture and OASIS, based on their clinical consequence and potential long-term sequala^4,19^. Individual neonatal outcomes included Apgar score < 4 and < 7 was added as individual outcomes, as diagnostic codes for neonatal outcomes might be underreported^20^.

There was no formal patient involvement in planning the study or interpretation of results, but selected outcomes were supported by key elements in the FIGO good practice recommendations counselling for vaginal birth after CS^4^.

### Statistical analyses

Characteristics of the study population were described according to IOL or expectant management at different gestational week strata: 37–38 (early term), 39–40 (full term), 41 (prolonged pregnancy), and > 41 weeks (post-term) and reported as frequencies and relative proportions or means with standard deviations according to data properties. The analysis emulated a sequence of daily target trials from 37 +0 to 42 weeks of gestation. The data were expanded into a person-day dataset, with gestational age expressed in days. Women entered the risk set at week 37 + 0 and contributed one observation for each gestational day on which they remained undelivered. On each day, women who were undelivered were classified as being managed expectantly (EM), whereas women for whom induction had been initiated were classified as undergoing IOL. Thus, women managed expectantly remained in the EM group until delivery, irrespective of whether the eventual delivery was vaginal or by caesarean section. Women undergoing IOL changed exposure status to IOL on the day induction was initiated. Women left the risk set on the day of delivery. For women with stillbirth, follow-up was censored three days before the registered date of birth; these women were classified as EM until censoring. The outcome represented the factual delivery outcome observed for the woman during follow-up. For example, a woman who remained under expectant management until 39 + 3 and underwent caesarean delivery contributed observations classified as EM on each preceding day, with caesarean delivery as the factual outcome. Her observations therefore contributed to estimation of the risk of caesarean delivery under expectant management at those gestational days. For each outcome, we fitted a logistic generalized additive model to the person-day data where gestational age was modelled continuously in days using a penalized regression spline, with the association between gestational age and the factual outcome allowed to differ according to whether women were undergoing IOL or remained under EM on that day. The model was specified as

$$\:\mathrm{logit}\left\{P\left({Y}_{it}=1\right)\right\}=\:{\beta\:}_{0}+{\beta\:}_{1}{IOL}_{ij}+{f}_{IOL}\left({t}_{ij}\right)\bullet\:I\left({IOL}_{ij}=1\right)+{f}_{EM}\left({t}_{ij}\right)\bullet\:I\left({EM}_{ij}=1\right)+\:{\beta\:}^{T}{X}_{i}$$

where $\:{Y}_{ij}$ is the factual outcome for woman * i* on person-day * t*, $\:{IOL}_{ij}$ indicates induction of labour on that day with EM as the reference, $\:{t}_{ij}$is gestational age in days and $\:{f}_{IOL}$and $\:{f}_{EM}$are penalized regression splines allowing the association between gestational age and the outcome to differ by mode of onset. The adjustment set comprised previous vaginal birth, pregestational disorders, gestational disorders, interpregnancy interval in months, maternal age, year of birth, country of birth, educational attainment, maternal weight, inverse squared maternal height, and their interaction (i.e., maternal BMI). These variables were selected a priori based on the authors’ theoretical framework regarding potential confounding and observed differences in maternal characteristics according to mode of onset.

For each gestational day, outcome risks under IOL and EM were estimated using model-based standardization. Predicted outcome probabilities were calculated for each observation under each mode of onset while retaining the observed values of the adjustment variables. These predictions were then averaged over the covariate distribution at each gestational day to obtain standardized, population-average risks under IOL and EM. Risk differences and risk ratios were derived from these standardized risks, with EM as the reference group. Figures display model-derived estimates across the full gestational-age range whereas tables present estimates at selected gestational ages.

To account for clustering of women within birth units and the repeated person-day observations contributed by each woman, uncertainty in the model-derived quantities was estimated using a two-stage non-parametric cluster bootstrap. At each bootstrap iteration, birth units were sampled with replacement from the observed birth units. Within each selected birth unit, women were sampled with replacement, and all person-day observations belonging to each sampled woman were retained. Thus, the within-woman dependence arising from the person-day expansion was preserved. The complete modelling and standardization procedure was then repeated for each bootstrap sample. A total of 250 bootstrap samples were generated, and 95% pointwise confidence intervals were obtained from the empirical bootstrap distribution of the model-derived quantities.

No formal a priori sample-size or power calculation was performed, and missing data were not imputed. Statistical analyses were performed using SPSS version 28 (IBM Corp., Armonk, NY, USA) and R version 3.6.3. A statistical analysis plan, defining the population, intervention, primary and secondary outcomes, statistical models, was formulated before analysis (table S1). We followed the STROBE initiative and checklist of reporting of cohort studies^21^.

All methods used in this study were performed in accordance with the relevant guidelines and regulations. All pseudonymized data handling complied with applicable ethical standards, legal requirements, and regulations governing the use of register-based health research. The Swedish Ethical Review Authority approved the study Jan 28 2020, number 2019–04925, with amendment March 17th 2022, Lund other division, number 2022-00922-02. The Swedish National Board of Health and Welfare approved the release of pseudonymized register data for research purposes under strict legal and data protection conditions. Informed consent is not required, as data are derived from mandatory national medical health registers collected under statutory obligation. The data are presented at an aggregated level.

## Results

For women with one prior CS, births registered as elective CS (*n* = 19 070), placenta previa, placenta accreta, preterm pre-labour rupture of membranes, or gestational length < 37 weeks (*n* = 2364) or congenital anomaly (*n* = 1405) were excluded; thus, 38 689 women intending a vaginal birth at term after CS were analysed (Figure S1). In the complete case analysis 35 812 births were included. Maternal and pregnancy characteristics according to IOL and expectant management at aggregated gestational weeks strata (37–38, 39–40, and ≥ 41) are presented in Table 1. Mean maternal age, height, weight, BMI, and interpregnancy interval differed between IOL and expectant management groups. At early term (37–38 weeks), 11.0% of women with IOL had a pre-gestational disorder, 27.4% a gestational disorder, 4.2% of neonates were SGA, and 13.2% LGA, reflecting a medical indication of induced labour. In prolonged and post-term pregnancies (≥ 41 weeks), 16.0% of women had a gestational disorder, 2.3% of neonates were SGA, and 3.2% LGA. Early term, women with IOL had a prior vaginal birth (41.7%) more often, compared with women with IOL in prolonged pregnancies and post-term (23.1%).

**Table 1:** Characteristics of births according to onset of labour at different gestational weeks (*n* = 38 689).

Gestational week	37–38^a^	> 38	39–40^b^	> 40	41^c^	> 41
	IOL	EM	IOL	EM	IOL	EM
N=	1612 n (%)	31 259 n (%)	3072 n (%)	11 096 n (%)	1581 n (%)	3024 n (%)
Age, years	32.8 [4.6]	32.8 [5.0]	33.0 [4.9]	32.8 [4.5]	33.1 [4.9]	32.8 [4.5]
Height, cm *Missing n= 1447*	165 [6.4]	165 [6.4]	165 [6.5]	166 [6.3]	166 [6.4]	166 [6.2]
Weight, kg *Missing n= 2293*	74 [16.0]	71 [14.5]	75 [16.7]	72 [14.8]	75 [16.4]	74 [15.4]
BMI (kg/m^2^) ≥ 30 *Missing n = 2311*	441 (29.2)	5408 (18.4)	1247 (28.2)	2113 (20.1)	386 (25.8)	657 (22.9)
Non-Nordic *Missing n= 33*	277 (17.2)	6130 (19.6)	636 (20.7)	2152 (19.4)	348 (22.0)	639 (21.1)
Co-habitant *Missing n= 1631*	1452 (94.1)	28 761 (95.7)	2793 (94.1)	10 181 (95.2)	1446 (94.5)	2731 (93.9)
Education > 12 years *Missing n= 754*	760 (47.8)	17 690 (57.7)	1613 (53.4)	6289 (57.8)	867 (56.3)	1654 (55.6)
IP interval, months	42 [35]	36 [30]	41 [35]	37 [30]	40 [33]	37 [30]
Prior vaginal birth	672 (41.7)	8389 (26.8)	963 (31.3)	2744 (24.7)	366 (23.1)	718 (23.7)
Pre-gestational disorders	178 (11.0)	968 (3.1)	226 (7.4)	267 (2.4)	55 (3.5)	69 (2.3)
Gestational disorders	442 (27.4)	2025 (6.5)	704 (22.9)	548 (4.9)	253 (16.0)	88 (2.9)
SGA *Missing n= 37*	68 (4.2)	476 (1.5)	77 (2.5)	154 (1.4)	37 (2.3)	41 (1.4)
LGA *Missing n= 37*	213 (13.2)	1186 (3.8)	218 (7.1)	310 (2.8)	50 (3.2)	85 (2.8)

Data are presented as mean [standard deviation] or number (percentage).

^a^ Women with spontaneous or CS onset 37–38 were excluded (*n* = 5818), ^b^ Women with spontaneous or CS onset 39–40 were excluded (*n* = 17,091), ^c^ Women with spontaneous or CS onset 41 weeks were excluded (*n* = 6491).

IOL: induction of labour, EM: expectant management, IP: interpregnancy interval, SGA: infant small for gestational age (−2 SD), LGA: infant large for gestational age (+ 2 SD).

Pre-gestational disorders: hypertension, diabetes mellitus, SLE, inflammatory bowel disorder, chronic kidney disorder, epilepsy.

Gestational disorders: hypertensive disorder of pregnancy, gestational diabetes, intrauterine growth restriction.

**Fig. 1:**
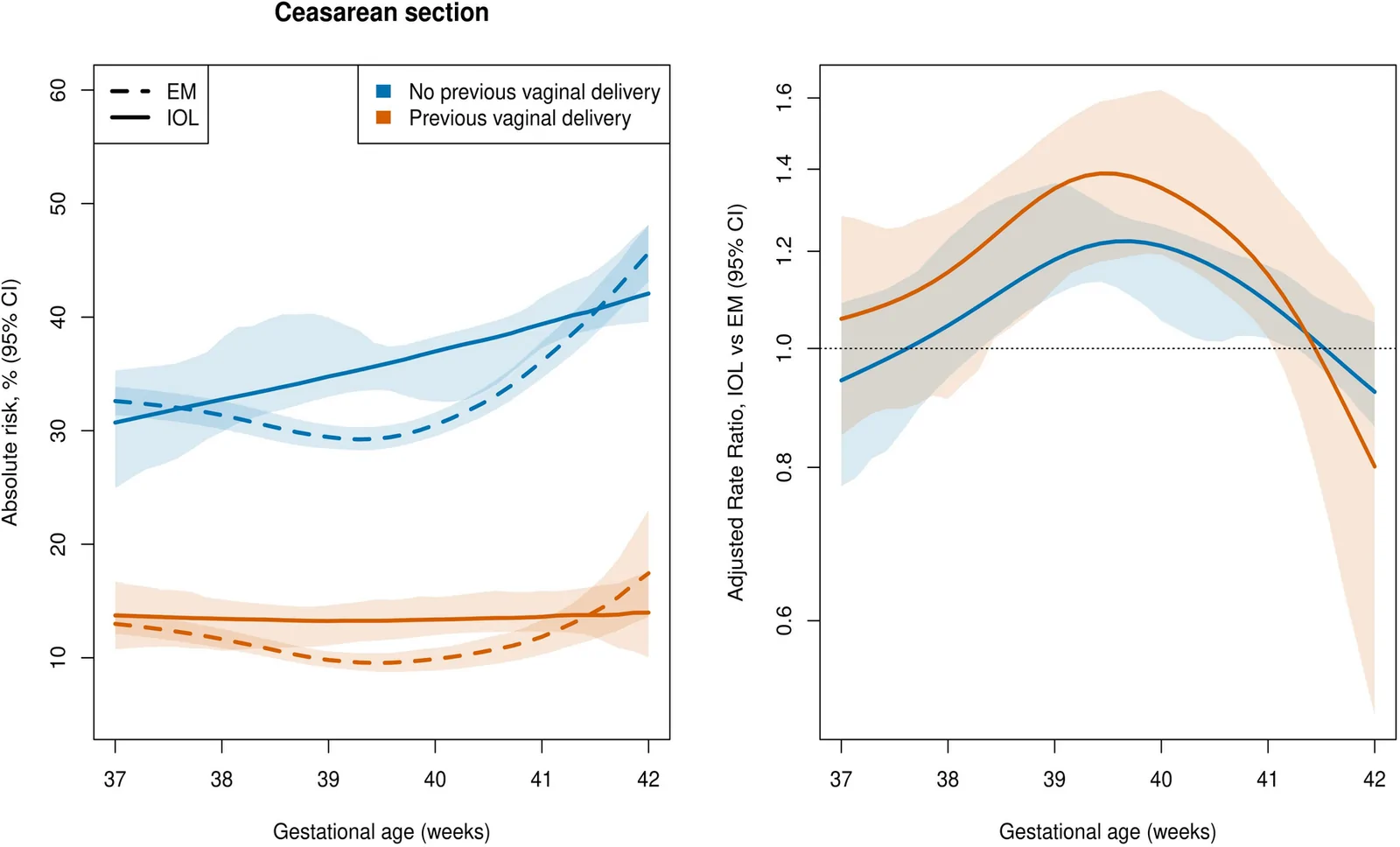
Adjusted absolute and relative risk ratios for caesarean section after induction of labour (IOL) compared with expectant management (EM) stratified by previous vaginal birth. Adjustments were made for: year of birth, maternal age, country of birth, education, interpregnancy interval, maternal height and weight with interaction, pre-gestational disorders, and gestational disorders.

The absolute adjusted risk for CS ranged from 25.5% (95% CI 21.5;29.9) with IOL at 37 weeks to 38.3% (95% CI 35.7;41.5) for the expectant management group at ≥ 42 weeks (Fig. 1, Table S2). Compared with expectant management, IOL increased the relative risk of CS at weeks 39, 40, and 41, but not in early-term or post-term pregnancies (Fig. 1, Table 3). The adjusted RD was 4.9% (95% CI 3.5;7.4) at 39 weeks, 5.5% (95% CI 3.0;6.6) at 40 weeks, and 2.9% (95% CI 0.9;4.9) at 41 weeks (Table 2). The absolute risk for CS for women without prior vaginal birth ranged from 31% with IOL at 37 weeks to 46% with expectant management at 42 weeks. For women with a prior vaginal birth, the rate of CS followed a similar pattern but was approximately 13–14% and stable for IOL over gestational weeks (Fig. 1, Table S2). The absolute risk for CS for women without medical conditions were lower than for women with medical conditions. Compared with expectant management IOL increased the risk of CS for women without medical conditions, but not for the group with medical disorders (Table S3, Fig S2).

**Table 2:** Risk difference (%) for maternal and neonatal outcomes 37–41 weeks after induction of labour compared with expectant management.

Gestational week		37			38			39			40			41		
	*N*=	*n*=	RD	95% CI	*n*=	RD	95% CI	*n*=	RD	95% CI	*n*=	RD	95% CI	*n*=	RD	95% CI
Caesarean section	10 958	1044	−0.5	−4.3;4.2	1994	1.8	−1.2;4.7	2069	**4.9**	3.5;7.4	2507	**5.5**	3.0;6.6	3344	**2.9**	0.9;4.9
No prior vaginal birth	18 552	813	−1.9	−7.4;2.9	1614	1.4	−1.6;7.0	1778	**5.3**	3.1;10.6	2236	**6.5**	1.6;7.8	3007	**3.3**	0.7;6.0
Prior vaginal birth	1510	231	0.7	−1.9;3.6	380	1.8	−1.1;3.4	291	**3.4**	1.2;4.9	271	**3.5**	2.0;5.5	337	**1.8**	0.2;4.2
Maternal outcome																
Severe composite ^a^	503	23	−0.0	−0.5;1.7	35	0.1	−0.4;0.5	98	0.1	−0.5;0.4	153	0.12	−0.4;0.3	194	0.1	−0.2;0.9
Uterine rupture	458	21	0.0	−0.5;1.4	30	0.1	−0.4;0.4	87	0.10	−0.5;0.5	144	0.1	−0.3;0.5	176	0.1	−0.2;1.1
Moderate composite ^b^	7707	329	0.9	−2.1;3.1	802	0.8	−2.0; 2.1	1560	0.35	−1.3;1.3	2462	−0.2	−1.1;1.6	2554	−0.64	−1.8;0.9
OASIS	1741	34	**−1.5**	**−2.6; −0.6**	148	**−1.2**	**−2.0; −0.5**	366	**−1.0**	**−1.6; −0.5**	606	**−0.8**	**−1.2; −0.2**	587	−0.4	−0.9;0.2
Neonatal outcome																
Severe composite ^c^	746	41	−0.1	−0.9;0.7	80	−0.1	−0.7; 0.34	136	−0.1	−0.6;0.2	236	−0.2	−0.6;0.6	253	−0.2	−0.7;0.4
APGAR < 4	180	14	0.2	−0.2;0.6	24	0.2	−0.2;0.4	32	0.2	−0.2;0.3	56	0.2	0.0;0.5	54	0.2	0.0;0.4
Moderate composite ^d^	1867	153	**3.8**	**1.4;7.4**	228	0.3	−0.6;1.1	339	**−1.0**	**−1.6; −0.3**	524	−0.9	−1.6; 0.1	623	−0.4	−1.3;0.7
APGAR < 7	729	56	0.6	−0.5;3.3	83	0.5	−0.4;1.0	128	0.3	−0.3;0.7	215	0.1	−0.2;0.8	247	0.0	−0.4;0.7

SN= numbers of events at each gestational week and for week 41 and onward. RD=adjusted risk difference (% with 95% CI) between IOL and EM.

Adjustments were made for the following confounders: year of birth, maternal age, country of birth, education, previous vaginal birth, interpregnancy interval, maternal height and weight with interaction, pre-gestational disorders, and gestational disorders.

^a^ maternal death (*n* = 1), re-operation (*n* = 47), or uterine rupture (*n* = 458). ^b^ blood loss > 1000 ml at birth or a diagnosis of anaemia at discharge (*n* = 4834), postpartum wound infection, hematoma, or dehiscence (*n* = 2469), or obstetric anal sphincter injury (OASIS) (*n* = 1741). ^c^ perinatal death (*n* = 83), neonatal birth trauma (*n* = 448), intracranial haemorrhage (*n* = 14), seizures/hypoxic encephalopathy (*n* = 100), necrotising enterocolitis (*n* = 3), or an Apgar score < 4 at five minutes (*n* = 180). ^d^ neonatal infection (*n* = 434), respiratory distress syndrome/aspiration (*n* = 1041) or Apgar score < 7 at five minutes (*n* = 729). Conditions can be overlapping.

Bold value indicates statistically significant.

**Fig. 2:**
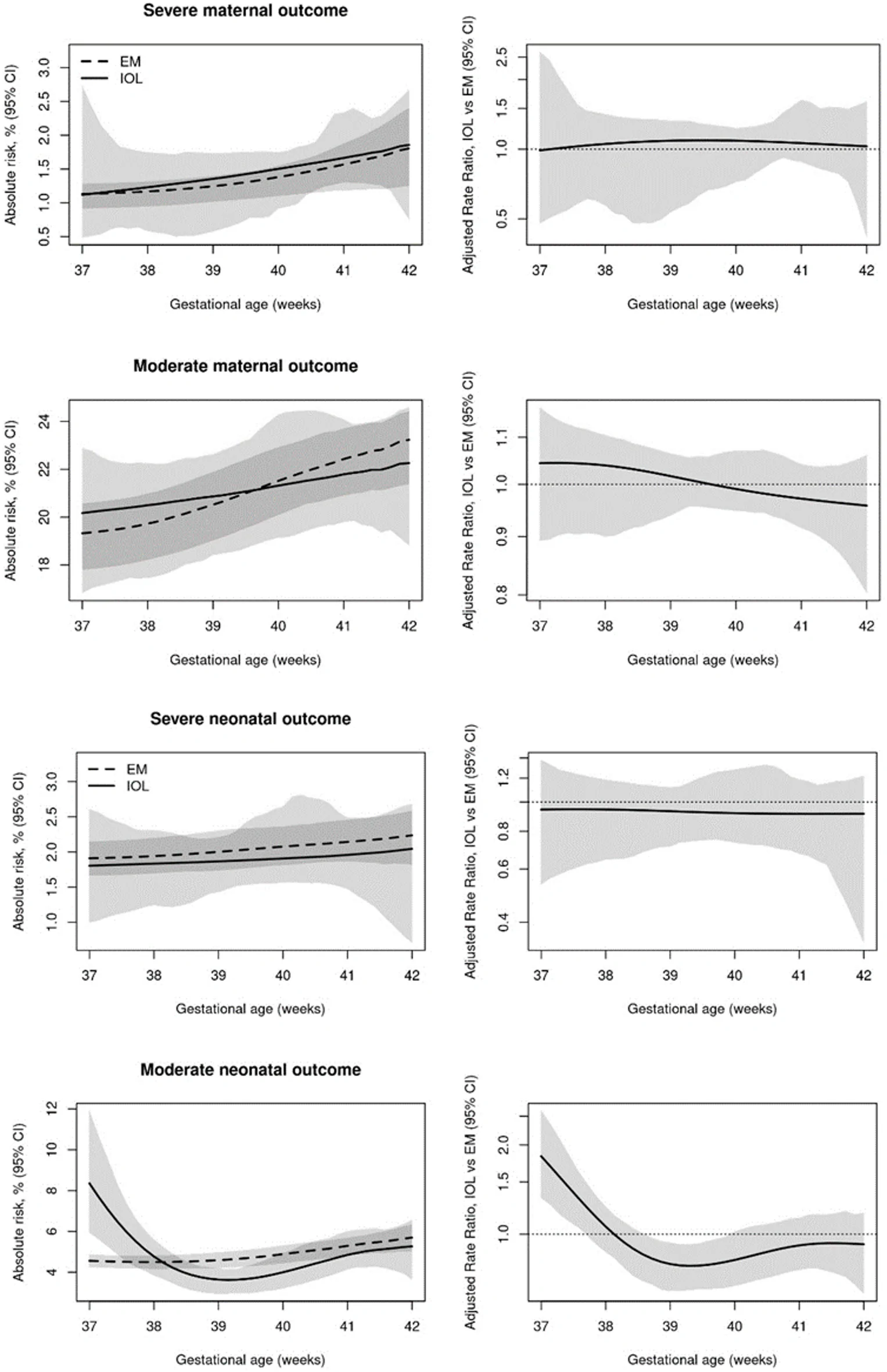
Adjusted absolute and relative risk ratios for maternal and neonatal morbidity after induction of labour (IOL) compared with expectant management (EM).Adjustments were made for: year of birth, maternal age, country of birth, education, prior vaginal birth, interpregnancy interval, maternal height and weight with interaction, pre-gestational disorders, and gestational disorders.

The absolute risk of severe maternal outcome increased from 1.1% to 1.9%, and severe neonatal outcome from 1.8% to 2.2%, from early term to post-term (*p* < 0.001) (Fig. 2, Table S2). There was no increased risk of severe maternal or neonatal composite outcomes with IOL compared with expectant management at any gestational week (Fig. 2; Table 3). Compared with expectant management, IOL increased the moderate neonatal composite outcome at week 37 (adjusted RD 3.8%, 95% CI 1.3;2.6) but reduced the risk at week 39 (adjusted RD −1.0%, 95% CI −1.6; −0.3) (Fig. 2; Table 2). The rate of uterine rupture after IOL ranged from 1.0% early term to 1.7% post-term. The corresponding rates were 1.0% and 1.6% with expectant management, and no significant increase in the risk of uterine rupture of IOL compared with expectant management was found at any gestational week (Fig. 2; Table 2). The absolute risk of OASI was lowest for IOL early term (2.1%, 95% CI 1.0;2.8), and the rate was twice as high with expectant management at 40 weeks onward. Compared with expectant management, IOL at week 37–40 reduced the risk of OASIS (Fig. 2; Tables 2 and 3).

In a sensitivity analysis, we excluded women with emergency CS before labour (*n* = 1417) and point estimates for failed vaginal delivery at different gestational weeks changed slightly but pointed in the same direction (data not presented).

**Table 3:**
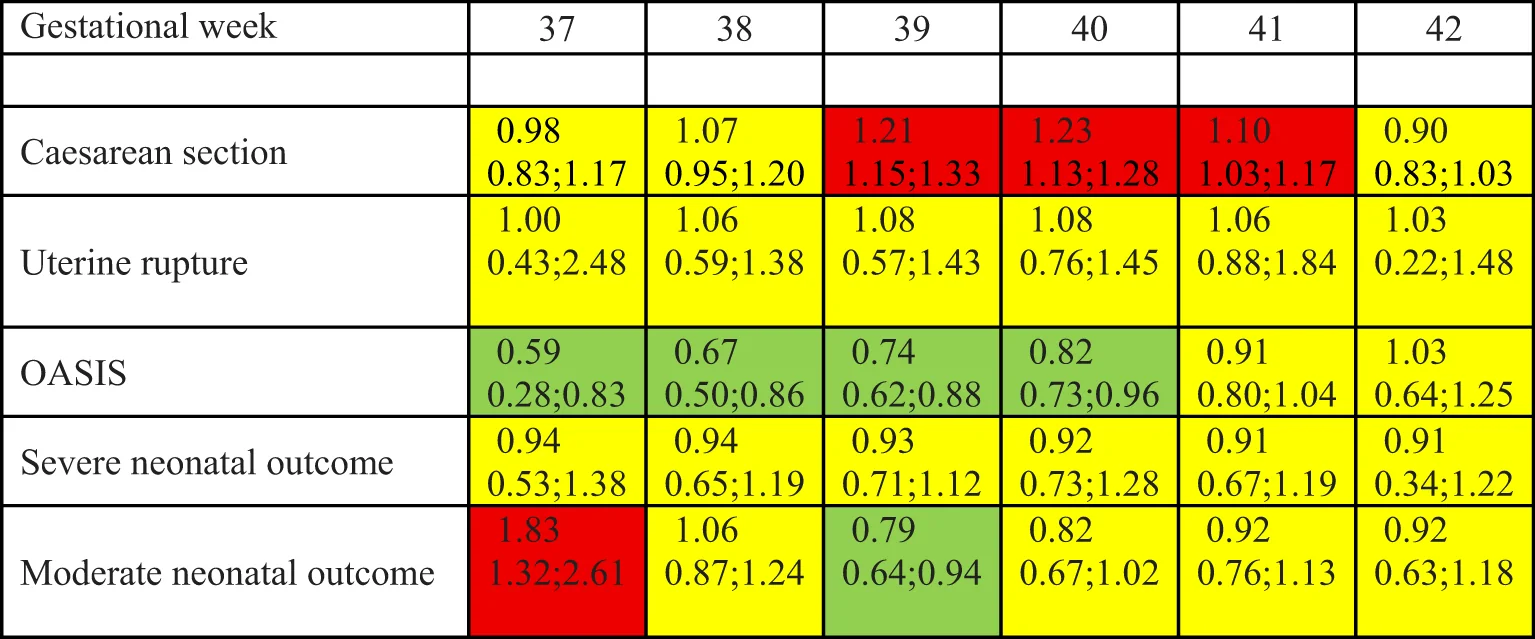
Relative risk of maternal and neonatal outcomes according to induction of labour (IOL) compared with expectant management (EM) at different gestational weeks strata.

(red=increased risk, yellow=neutral, green=reduced risk) Adjusted relative risks with 95% CI; year of birth, maternal age, country of birth, education, previous vaginal birth, interpregnancy interval, maternal height and weight with interaction, pre-gestational disorders, and gestational disorders. Obstetric anal sphincter injuries (OASIS). Severe neonatal outcome: perinatal death, neonatal birth trauma, intracranial haemorrhage, seizures, hypoxic encephalopathy, necrotizing enterocolitis, or an Apgar score <4 at five minutes Moderatae neonatal infection, respiratory distress syndrome, aspiration, or Apgar score <7 at five minutes.

## Discussion

### Main findings

In women with one previous CS, induction of labour (IOL) was associated with increased risk of CS at full term (week 39–41), but not early term (week 37–38) or post-term (week ≥ 42), compared with expectant management. The rates of both maternal and neonatal severe outcomes and uterine rupture increased with gestational age, but there was no risk difference between IOL and expectant management. Moderate neonatal outcome was increased with IOL at week 37 but reduced at week 39, and IOL at week 37 to 40 reduced OASIS compared with expectant management.

### Strengths and limitations

The study was based on prospectively collected data from the Swedish MBR, which has excellent coverage and high validity of registered reproductive data at a population-based level^20^. Information on onset of birth is routinely registered in check boxes by midwives, and missing values are negligible. The study was designed to mimic a clinical situation: exposure was based on the mode of onset, not the mode of birth, and compared with expectant management using remaining individuals each day of gestation. This is important since the assignment to onset of labour can only be IOL, planned CS or expectant management. Our study population was restricted to women with one prior CS, with a singleton pregnancy without diagnoses of placenta previa or accreta, who would have been eligible for a trial of labour at term in clinical practice, however we did not restrict our study to low-risk pregnancies. The large size of the cohort enabled us to investigate rare but important outcomes, such as uterine rupture, and to investigate outcomes at a continuum of gestational length in days.

A major limitation is that we had no information about indication of index CS, such as arrest disorders, which may have affected consultations regarding the planned mode of delivery (trial of labour or elective CS) and the chance of successful vaginal birth^22^.Another caveat is that, despite excluding pre-labour CS registered as elective, we cannot rule out that women scheduled for a planned CS with a spontaneous onset before the day of surgery were included in the study population as intrapartum CS after spontaneous onset. This could introduce a misclassification bias, as they will be more commonly represented in the expectant management group early term, and could possibly explain the reduced relative risk of CS observed with IOL early term. Important information was lacking about the time of onset for pregnancy complications indicating IOL and their severity, which limited opportunities to fully balance groups in terms of confounding by indication. Also, no information on gestational length at the onset of labour (either spontaneous or induced) was available, but only gestational age at birth. This is not in accordance with the situation in a RCT and, potentially introduce information bias. This could imply that the onset of labour occurred one or several days earlier, and the figures show a rightward shift, with the breakpoint for no increased risk of CS with IOL vs. expectant management occurring at 41 weeks. We had no information on duration of labour, however this was investigated in another Swedish sample of women with prior CS, where the mean time from established contractions to birth was estimated to be 8 h for women with vaginal birth and 11 h for women with CS^23^

Furthermore, we had no information of cervical status at the start of induction and we did not analyse methods of IOL separately, which could have influenced the rates of intrapartum CS. In Sweden, the national clinical guideline proposes cervical catheter as the primary method for IOL in women with previous CS, and not prostaglandins for safety reasons. Apart from electronic heart monitoring, there is no national consensus of acceptable non-medical indications for IOL or labour management such as maximum doses of oxytocin for women attempting vaginal birth after CS. Outcomes were based on register data with limited granularity; for example, postpartum infections were based on diagnoses from specialised care until 42 days postpartum, but not primary care and not antibiotic prescriptions, which could explain the low rates and do not give the complete picture of postpartum complications for women undergoing a trial of labour after CS.

### Interpretation

Women undergoing a trial of labour after CS face challenges and an increased risk of negative birth-related outcomes compared with women with previous vaginal births^1,3^. Prior CS is a strong risk factor for severe maternal morbidity, including maternal mortality, uterine rupture, massive bleeding^2,24^, and neonatal morbidity^2,25^. In a systematic review focusing on maternal and neonatal outcomes of labour after prior CS, elective CS compared with a trial of labour was associated with increased maternal mortality (0.13‰ vs. 0.04‰), but reduced neonatal mortality (0.05% vs. 0.13%)^5^. In our study, CS registered as elective were excluded, and the population consisted of women with an intended vaginal birth, in which women with a birth after IOL were compared with the remaining women each given day. The rates of severe maternal and neonatal outcomes were low (1–2%). The main contributor to severe maternal outcome was uterine rupture, a feared complication after CS^4,26^. The adjusted absolute risk for uterine rupture and severe maternal or neonatal outcome increased with gestational age, but with no risk difference between IOL and expectant management. This contrasts with numerous observational studies that have identified IOL as a risk factor for uterine rupture^14,23^. However, many of these studies have compared IOL with spontaneous onset, rather than expectant management. Although there is a lack of RCT: s comparing IOL with expectant management for women with prior CS, there are other cohort studies with a similar design investigating IOL versus expectant management, defined as birth the subsequent week and onward^14,28^. In contrast to Palatnik et al. (*n* = 12 576)^14^ and Stock et al. (*n* = 46 176)^29^, we found an increased rate of repeat CS after IOL at term, with an adjusted absolute risk difference of 2.9% to 5.5% compared with EM. Despite the attempted adjustments, the groups IOL and EM may be unbalanced in terms of underlying conditions and prior obstetric history, that could explain the higher rates of CS observed after IOL at term. At early term, there might be a selection of women with a favourable cervical status into the IOL group, which could explain the lower risk.

The absolute risk for CS for women with no prior vaginal birth ranged from 31% with IOL at 37 weeks to 46% with expectant management at 42 weeks, which means that a primary CS instead of IOL or expectant management could be a reasonable option at later gestational weeks. The absolute risk for women with a prior vaginal birth was, as expected, lower and more stable over weeks. The absolute risk for CS for women without medical conditions were lower than for women with medical conditions, but compared with expectant management the risk of CS was only increased for women without medical conditions.

Lappen et al. reported similar results to our study, regarding failed attempt of labour at week 37–39, but not at later gestations, and no increased risk of severe neonatal morbidity, when comparing IOL with expectant management^28^. In contrast, they observed an increased risk of severe maternal morbidity with IOL at 39 weeks of gestation compared with expectant management, including either maternal mortality, hysterectomy, ICU admission, thrombosis, or transfusion. The rate of adverse maternal composite ranged from 1.2% to 5.3% at different gestational weeks, compared to our rate of severe composite from 1.1% to 1.8%. However, different definitions of adverse outcomes and methods for data collection restrict direct comparisons of results. Stock et al. found an increased likelihood of neonatal admission after IOL at 39 weeks. In contrast, but in accordance with the ARRIVE trial^9^, we found a reduced risk of a composite of moderate neonatal outcomes after IOL at 39 weeks.

## Conclusions

As global CS rates have risen over the last decades, increased knowledge of appropriate clinical management and counselling in the subsequent birth is essential. This includes information on potential risks and benefits of medical interventions such as induced labour. Among women with prior caesarean section, we observed an increased risk of repeat CS with IOL at week 39–41, but not early term or post-term or for women with pre-gestational or gestational disorders, compared with expectant management. The benefits of IOL included reduced rates of OASI and reduced risk of moderate neonatal morbidity at week 39. There was no risk difference of IOL or expectant management for severe maternal or neonatal morbidity; however, these rare outcomes increased steadily with gestational length, which could be considered in the timing of birth.

There is currently an urge for RCT: s including women with prior CS. Although such trials may not be sufficiently powered to detect differences in rare outcomes, the success or failure of vaginal birth can be assessed. Furthermore, shared individual patient data analysis from different trials would permit a more robust conclusion regarding the risks and benefits of timing and induction of labour in this group.

## Supplementary Information

Below is the link to the electronic supplementary material.

Supplementary Material 1

Supplementary Material 2

## Data Availability

Medical birth register data that support the findings of this study are available from Swedish National board of Health and Welfare, but restrictions apply to the availability of these data, which were used after Ethical approval from the Swedish Ethical Review Authority for the current study and so are not publicly available.
