# Supplementary material for: Induction of labour versus expectant management after one previous caesarean birth maternal and neonatal outcomes in a population-based cohort study
Source: Sci Rep. 2026 Sep 9;16:28237. doi: 10.1038/s41598-026-70966-9 (PMC13558596; doi:10.1038/s41598-026-70966-9)
Supplement: Supplementary file 1 [file 41598_2026_70966_MOESM1_ESM.pdf]

Table S1. Target Trial Emulation of Induction of Labor versus Expectant Management.

**Protocol component**	**Target trial**	**Emulation using observational data**
**Eligibility**	Pregnant women at start of gestational day X who:<br>• Are undelivered<br>• Have reached 37+0 weeks<br>• Are clinically eligible for both induction (IOL) and expectant management (EM)	For each gestational day X, construct risk sets of women still pregnant and meeting eligibility criteria
**Treatment strategies**	• Initiate induction on day X<br>• Expectant management (no induction on day X)	At each day X, classify exposure:<br>• Induction initiated<br>• No induction that day<br><br>Women may enter sequential trials daily until delivery.
**Assignment**	Randomization at start of day X to one of the two strategies. Women are aware of their assigned treatment	Assume conditional exchangeability given baseline covariates and gestational day
**Time zero**	Start of gestational day X	Person-day data, time zero defined daily.
**Follow-up**	From day X until outcome occurrence or end of follow-up.	Same as target trial. Women exit after delivery. Pregnancies ending in stillbirth are administratively censored 3 days prior to recorded delivery date to account for uncertainty in timing of fetal demise.
**Outcomes**	Maternal and neonatal outcomes	Same as target trial
**Causal contrasts**	For each gestational day, compare IOL to EM	Marginal risk differences (RD) and relative risks (RR) comparing risks under IOL and EM. Risks standardized over the empirical distribution of baseline covariates and gestational day using g-computation

**Analysis plan**	Compare randomized groups	1) Fit adjusted pooled logistic model for each outcome including:<br>a) Indicator for IOL with EM as reference<br>b) Baseline covariates<br>c) Interactions between gestational day spline with treatment indicators<br>2) Use g-computation to predict risks under each strategy<br>3) Compute marginal RD and RR comparing IOL vs EM.<br>4) Use the cluster bootstrap (resampling hospitals and women within hospitals) for 95% confidence intervals

Figure S1. Flowchart study population.

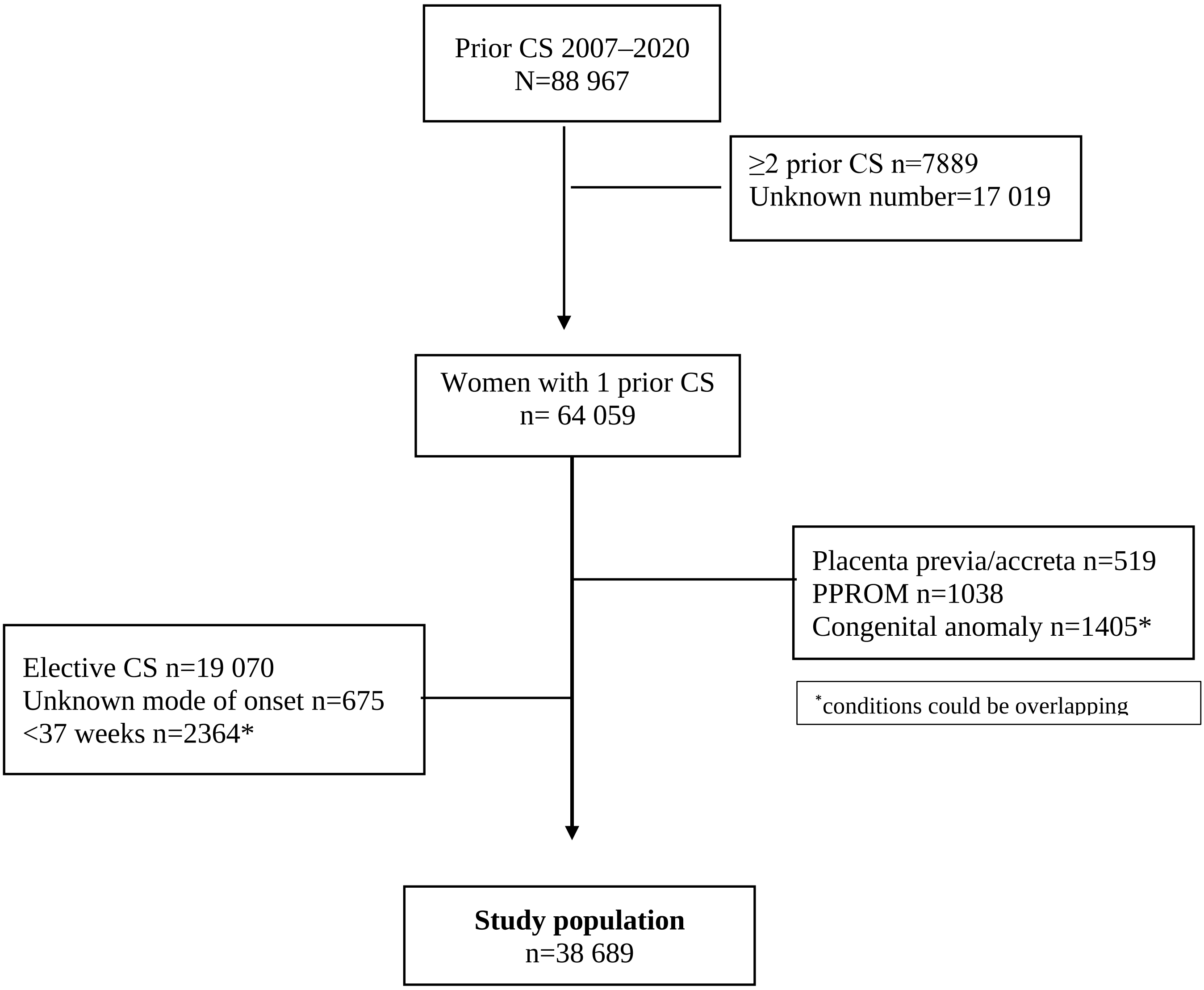

Table S2. Absolute adjusted risks for **maternal and neonatal outcomes** after IOL and expectant management at different gestational ages.

Gestational week	37		38		39		40		41		42	
	**IOL**	**EM**	**IOL**	**EM**	**IOL**	**EM**	**IOL**	**EM**	**IOL**	**EM**	**IOL**	**EM**
**Maternal outcomes**												
**CS**	25.5	25.8	26.5	24.6	27.6	22.7	29.4	23.8	31.6	28.7	34.5	38.3
95% CI	21.5;29.9	24.3;27.1)	23.6;29.2	23.1;25.8	26.0;30.3	21.5;23.7	26.6;30.1	22.7;24.8	29.6;33.1	27.2;30.1	31.5;39.7	35.7;41.5
No prior vaginal birth	30.7	32.6	32.7	31.4	34.8	29.4	37.0	30.5	39.4	36.1	42.1	45.6
95% CI	24.9;35.3	31.4;33.9	29.9;38.1	30.1;32.6	33.0;40.0	28.4;30.1	32.6;38.3	29.5;31.6	37.3;41.8	34.6;37.9	39.6;48.1	43.1;48.1
Prior vaginal birth	13.7	13.0	13.4	11.6	13.2	9.8	13.4	9.9	13.6	11.8	14.0	17.4
95% CI	10.8;16.7	12.1;14.0	10.6;15.2	10.8;12.6	11.1;14.6	9.1;10.6	12.0;15.3	8.9;10.9	12.3;15.9	10.3;13.3	10.1;17.5	13.6;23.0
**Severe composite**[a]	1.1	1.1	1.2	1.2	1.4	1.3	1.5	1.4	1.7	1.6	1.9	1.8
95% CI	0.5;2.7	0.9;1.3	0.6;1.8	1.0;1.3	0.6;1.7	1.0;1.4	0.9;1.8	1.1;1.6	1.3;2.3	1.2;1.9	0.8;2.7	1.3;2.4
**Moderate composite**[b]	20.2	19.3	20.5	19.7	20.9	20.5	21.3	21.5	21.8	22.4	22.3	23.2
95% CI	16.8;22.9	17.8;20.6	17.5;22.3	18.2;21.0	18.4;22.6	19.0;21.9	19.1; 24.3	20.1; 22.9	19.8; 24.1	20.9; 23.7	18.8; 24.4	21.4; 24.6
Uterine rupture	1.0	1.0	1.1	1.1	1.2	1.1	1.4	1.3	1.5	1.4	1.7	1.6
95% CI	0.4;2.4	0.8;1.2	0.5;1.5	0.9;1.2	0.6;1.6	0.9;1.3	0.9;1.8	1.0;1.4	1.1;2.4	1.1;1.6	0.3;2.2	1.2;2.0
OASIS	2.1	3.6	2.5	3.7	3.0	4.0	3.5	4.2	3.9	4.3	4.2	4.3
95% CI	1.0;2.8	3.3;3.9	1.9;3.1	3.4;4.1	2.5;3.5	3.6;4.4	3.0;4.2	3.8;4.7	3.3;4.5	3.8;4.7	3,6;4.6	2.6;5.2
**Neonatal outcomes**												

Severe composite [c]	1.8	1.9	1.8	1.9	1.9	2.0	1.9	2.1	2.0	2.1	2.0	2.2
95% CI	1.0; 2.6	1.7; 2.2	1.3; 2.3	1.7; 2.2	1.4; 2.2	1.7; 2.3	1.6; 2.6	1.8; 2.4	1.5; 2.5	1.9; 2.5	0.7; 2.7	1.8; 2.6
Moderate composite [d]	8.4	4.6	4.8	4.5	3.6	4.6	4.0	4.9	4.9	5.3	5.3	5.7
95% CI	5.9;11.9	4.3; 4.9	3.8; 5.6	4.2; 4.8	2.9; 4.2	4.2;5.0	3.2;5.0	4.4; 5.3	4.1; 6.0	4.8; 5.7	3.6; 6.7	5.0; 6.3

Adjustments were made for the following confounders: year of birth, maternal age, country of birth, education, previous vaginal birth, interpregnancy interval, maternal height and weight with interaction, pre-gestational disorders, and gestational disorders.

[a] maternal death, re-operation or uterine rupture.

[b] blood loss >1000 ml at birth or a diagnosis of anaemia at discharge, postpartum wound infection, hematoma, or dehiscence, or obstetric anal sphincter injuries (OASIS).

[c] perinatal death, neonatal birth trauma, intracranial haemorrhage, seizures, hypoxic encephalopathy, necrotizing enterocolitis, or an Apgar score <4 at five minutes

[d] neonatal infection, respiratory distress syndrome, aspiration, or Apgar score <7 at five minutes
